# The Effect of a Citrus and Pomegranate Complex on Physical Fitness and Mental Well-Being in Healthy Elderly: A Randomized Placebo-Controlled Trial

**DOI:** 10.1007/s12603-022-1834-4

**Published:** 2022-08-06

**Authors:** Sanne Ahles, I. Cuijpers, F. Hartgens, F.J. Troost

**Affiliations:** 1 Department of Nutrition and Movement Sciences, School of Nutrition and Translational Research in Metabolism (NUTRIM), Maastricht University, 6200 MD, Maastricht, The Netherlands; 2 BioActor BV, Maastricht, The Netherlands; 3 Centre for Healthy Eating and Food Innovation, Division Food Innovation and Health, School of Nutrition and Translational Research in Metabolism (NUTRIM), Maastricht University, Venlo, The Netherlands; 4 Departments of Epidemiology and Surgery, Research Institute CAPHRI, Maastricht University Medical Centre, Maastricht, The Netherlands; 5 Sports Medicine Center Maastricht*Parkstad, Maastricht, The Netherlands

**Keywords:** Citrus fruit, polyphenols, hesperidin, physical fitness, quality of life

## Abstract

**Objectives:**

This study investigates whether a citrus and pomegranate complex (CPC) improves physical fitness, mental well-being, and blood biomarkers for oxidative stress and endothelial function in healthy elderly.

**Design:**

A randomized placebo-controlled cross-over trial.

**Participants:**

The study included 36 healthy elderly aged 60–75 years old.

**Intervention and Measurements:**

Participants received four weeks of CPC supplementation and performed the handgrip strength and senior fitness test. Quality of life (QOL) was assessed and blood samples were analyzed for oxidative stress and endothelial function markers.

**Results:**

After four weeks of CPC supplementation, handgrip strength significantly improved (p=0.019), compared to placebo. Moreover, the thinking, memory, learning, and concentration facets were improved (p=0.042), compared to placebo, and plasma malondialdehyde decreased, compared to placebo (p=0.033). The intervention did not affect senior fitness and the other QOL domains and blood parameters.

**Conclusion:**

Four weeks of daily CPC supplementation significantly improves handgrip strength and self-evaluated measures of psychological function in healthy older adults. Further research should focus on mechanisms associated with physical performance.

## Introduction

**T**he life expectancy in Western countries is increasing rapidly. The World Health Organization has predicted that by 2050, 16% of the total world's population will be aged 65 years and older (1). As ageing is considered a major risk factor for a variety of diseases such as cardiovascular disease, cognitive decline, and decreased physical function, an increasing number of individuals is dealing with a variety of age-related diseases (2, 3). Consequently, it is of importance to prevent the development of these diseases by maintaining physiological function and well-being at an older age, referred to as healthy ageing. Daskalopoulou et al. (4) reported in a systematic review that physical activity is positively associated with healthy ageing. Moreover, other modifiable factors related to a healthy lifestyle, such as smoking and drinking status, and leisure time activities, have also been shown to contribute to improved mental well-being and quality of life in elderly (5, 6). Therefore, maintaining a healthy lifestyle could be an important strategy to support healthy ageing and prevent these age-related pathologies (7).

Next to physical activity, nutritional status is an important lifestyle-related factor that has a substantial impact on healthy ageing (8). For example, diets high in fruits, vegetables, and fish are associated with longevity (9). Dietary supplementation with polyphenols has been suggested as a promising approach to prevent the negative consequences of ageing and to sustain a healthy lifestyle, before the onset of age-related comorbidities (10, 11). Moreover, four weeks of a high polyphenol diet resulted in improved physical- as well as mental health in hypertensive middle-aged adults (12). Hesperidin and punicalagins are polyphenols that are mainly found in citrus fruits and pomegranates, recognized for anti-inflammatory and antioxidant effects among others (13, 14). In previous randomized controlled trials, polyphenol supplementation has shown to decrease malondialdehyde (MDA) concentrations (15, 16), and to increase trolox equivalent antioxidative capacity (TEAC) (17), as well as nitrate levels, a marker for endothelial function (18). Moreover, beneficial effects of supplementation with these polyphenols on exercise performance have been observed in healthy adults and athletes (19, 20). However, to our knowledge, intervention studies using a combination of a citrus and pomegranate complex (CPC) in untrained older individuals have not been performed before. Therefore, the primary objective of this study was to investigate whether four weeks of supplementation with CPC improves physical fitness in healthy elderly, with secondary objectives to assess the effects of CPC on mental well-being and on systemic parameters of oxidative stress and endothelial function.

## Materials and Methods

The study was approved by the Medical Ethics Committee of the Maastricht University Medical Centre+ and was conducted from June 2018 to January 2019 in full accordance with the principles of the Declaration of Helsinki as amended in 2013 (Fortaleza, Brazil), and the Dutch Regulations on Medical Research involving Human Subjects (1998). All participants provided written informed consent before participation. This trial was registered at ClinicalTrials.gov under NCT03781999.

### Participants

Healthy adults aged 60 – 75y, and with a BMI between 18 and 28 kg/m^2^ were recruited through local media advertisements. Key exclusion criteria were: smoking; allergy to test product or citrus fruits; recent muscle injury before the start of the study; administration of dietary products containing citrus flavonoids or metabolites; supplement use; inability to perform the fitness tests; high blood pressure (systolic ≥ 140 mmHg, diastolic ≥ 90 mmHg); use of beta-blockers; abuse of alcohol (>20 alcoholic U/wk) or recreational drugs; and presence of medical conditions such as cardiovascular disease, and type 2 diabetes mellitus.

### Study Design and Protocol

The study was designed as a randomized, double-blind, placebo-controlled, cross-over trial to investigate the effects of a daily dose of citrus and pomegranate complex (CPC) versus placebo on physical fitness and quality of life in elderly. The study consisted of two randomized intervention periods (CPC and placebo) of four weeks, with a wash-out period of at least four weeks (with a maximum of eight weeks) (median: 4 weeks) in between. Before the start of the study, a familiarization test was conducted. Subsequently, test days took place before and after each four week intervention period. Test days comprised anthropometric measurements, venous blood collection after an overnight fast, a standardized breakfast, the WHO-QOL-100 questionnaire, the handgrip strength test, and the senior fitness test as detailed below. A randomization list was generated by an independent researcher with Research Randomizer (http://randomizer.org). Researchers involved in the execution of the study were independently Good Clinical Practice (GCP) certified and received additional training where necessary.

Participants were instructed to abstain from high intensity resistance exercise and consumption of caffeine and alcohol 24 hours prior to each test day. During the supplementation period, participants were instructed to refrain from foods high in citrus flavonoids (oranges, lemons, pomegranate and grapefruit) and to maintain their weekly training schedule and habitual diet.

#### Physical Fitness Tests

Each test day, handgrip strength was measured using the Hydraulic Hand Dynamometer, SH5001 (Saehan, Gyeongsangnam-do, S. Korea). The participants were requested to sit on a chair with their elbow by the side of their body, unsupported and flexed in an angle of 90 degrees with the wrist in a natural position (21). Subsequently, subjects squeezed the handle as hard as possible for five seconds. Handgrip strength was measured in triplicate for the dominant hand in kg, using the best attempt for further analysis.

Physical fitness was assessed using the senior fitness test, a validated test battery for older adults. This test comprises six measurements: (1) Chair Stand Test for lower body strength; (2) Biceps Curl Test for upper body strength; (3) Six-Minute Walk Test (6-MWT) for aerobic capacity; (4) Chair Sit and Reach Test for lower body flexibility; (5) Back Scratch Test for upper body flexibility and (6) Up and Go Test for agility (22). In the Chair Stand Test, participants were instructed to stand up from a chair and sit down again with arms folded on their chest, as many times as possible during a time frame of 30s. The Biceps Curl Test was performed using a weight of 2.5 kg for women and 4.0 kg for men. The amount of completed curls in 30s was registered for the dominant hand. For the 6-MWT, participants were instructed to continuously walk back and forth in a 20m corridor for six minutes, after which total walking distance (m) was calculated. In the Chair Sit and Reach test, participants were instructed to sit at the edge of a chair with their dominant leg extended and to reach for their toes. Distance between their fingers and toes was determined (cm) in triplicate. For the Back Scratch Test, participants had to bring their hands together behind their back by reaching over the shoulder with one hand, and under the shoulder with the other hand. The distance between the middle fingers was determined in triplicate. In the Up and Go Test, participants were instructed to stand up from a chair, walk 2.45m, and return to the seated position as fast as possible. For all tests, best attempts were used for analysis.

#### WHO-QOL-100 Questionnaire

The WHO-QOL-100 questionnaire was used to determine quality of life of the participants on each test day. The questionnaire contained 100 questions, covering 24 facets. (23). From these facet scores, the domain scores of physical capacity, psychological capacity, social relationships, environment, independence and spirituality were calculated and used for further analysis. Furthermore, facets were evaluated only if results on the domains showed a trend or a significant time*treatment interaction.

#### Blood marker analysis

Blood samples were taken in a fasting state on each test day. Blood samples were collected in Heparin S-Monovette tubes (Sarstedt, Nümbrecht, Germany). Tubes were immediately centrifuged at 3000 rpm at 4 °C for plasma samples and at room temperature for serum samples. Hereafter, aliquots were snap-frozen in liquid nitrogen and stored at −80 °C for subsequent analysis.

Plasma MDA concentrations were determined as oxidative stress biomarker in blood plasma using the Thiobarbituric Acid Reactive Substances (TBARS) assay (TBARS Assay Kit — Malondialdehyde Quantification, Bioquochem, Spain) (24). TEAC was measured in blood plasma using the Cupric Reducing Antioxidant Capacity (CUPRAC) assay kit (CUPRAC Antioxidant Capacity Assay Kit, Bioquochem, Spain) as a measure for antioxidant capacity (25). Plasma nitrate (NO3) concentrations, which reflect endothelial function, were measured by assessing reduction of NO3 to gas using the gas-phase chemiluminescence technique as previously described (26). The reaction of reduced NO with ozone results in luminescence which was detected by an NOanalyzer.

### Study product

Participants ingested two gelatin capsules per day containing 350 mg CPC (Actiful®, BioActor BV, Maastricht, The Netherlands), resulting in a total daily intake of 500 mg of a citrus sinensis extract (90% hesperidin, 450 mg), combined with 200 mg of a pomegranate fruit concentrate. This dose translates to approximately 0.9L of orange juice per day (27). Maltodextrin capsules (Gonmisol, Barcelona, Spain) were used as placebo and were identical to the study product in appearance and flavor due to the use of opaque capsules. Preparation of the capsules was performed by an independent researcher to ensure blinding. The capsules were consumed before breakfast with 200 mL of water. The daily dose of CPC was chosen based on previous studies showing a positive effect on exercise performance (20, 28).

### Statistical Analysis

Sample size was calculated using a significance level of α = 0.05 and a power of β = 0.80, with an effect size of 0.39 based on the Six-Minute Walk Test (29). In total, 36 participants were required to obtain sufficient power. Statistical analysis was performed using IBM SPSS Statistics (version 26.0, IBM Corporation, Armonk, NY, USA). Data were reported as estimated mean ± standard error of the mean (SEM). Intention to treat linear mixed model analyses were carried out using period, treatment, and time as fixed factors, and time*treatment as the interaction term. If the interaction term was not significant, it was removed from the model. For all analyses, two sided p-values < 0.05 were considered to be statistically significant.

## Results

**Figure 1:**
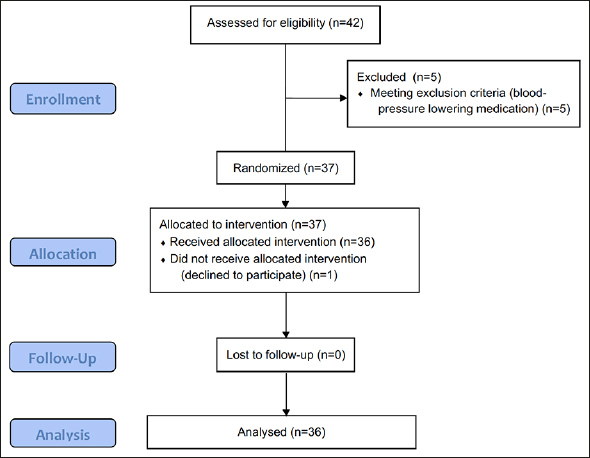
CONSORT flow diagram of the study participants

In total, 42 participants were assessed for eligibility, of which 37 were randomized. Five participants were excluded due to use of blood-pressure lowering medication. One participant dropped-out before receiving the allocated intervention, due to personal reasons. All remaining 36 participants completed the study and were included in the analyses. The mean age of the participants that finished the study was 66 ± 1 years, and consisted of 9 males and 27 females. Their average BMI was 25.3 ± 0.3 kg/m2, with a waist-hip-ratio of 0.90 ± 0.01. The CONSORT flow diagram is displayed in Figure 1.

### Physical Fitness

Outcomes of the physical fitness tests are reported in Table 1. A significant time*treatment interaction was observed for handgrip strength (p = 0.019). After four weeks of CPC supplementation, a significant improvement was observed on handgrip strength, compared to placebo.

**Table 1:** Outcomes of the physical fitness tests after four weeks of CPC or placebo supplementation^1^

**Physical Fitness Tests**		**CPC (n=36)**	**Placebo (n=36)**	Time*treatment interaction	**Main effect of time**	**Main effect of treatment**
	Handgrip strength (kg)			0.019^a^*	0.192^a^	0.280^a^
	Baseline	31.8 ± 1.7	31.9 ± 1.8			
	4 weeks	32.0 ± 1.6	31.0 ± 1.6			
SFT	Chair stand test (repeats)			0.160^a^	0.034^b^*	0.919^b^
	Baseline	15.6 ± 0.5	15.7 ± 0.6			
	4 weeks	16.1 ± 0.5	15.8 ± 0.6			
SFT	Biceps curl (repeats)			0.645^a^	0.009^b^*	0.090^b^
	Baseline	19.3 ± 0.5	19.8 ± 0.6			
	4 weeks	19.9 ± 0.5	20.3 ± 0.6			
SFT	Six-minute walk test (m)			0.361^a^	0.990^b^	0.901^b^
	Baseline	571.6 ± 8.1	576.1 ± 8.9			
	4 weeks	574.3 ± 8.8	573.4 ± 9.3			
SFT	Chair sit and reach test (cm)			0.906^a^	0.801^b^	0.479^b^
	Baseline	−5.9 ± 1.7	−5.1 ± 1.9			
	4 weeks	−6.0 ± 1.7	−5.1 ± 1.7			
SFT	Back scratch test (cm)			0.096^a^	0.235^b^	0.332^b^
	Baseline	6.3 ± 2.0	8.0 ± 2.2			
	4 weeks	6.5 ± 2.0	6.9 ± 2.1			
SFT	Up and go test (s)			0.996^a^	0.006^b^*	0.376^b^
	Baseline	4.7 ± 0.1	4.8 ± 0.1			
	4 weeks	4.6 ± 0.1	4.7 ± 0.1			


1. Data are presented as actual mean ± SEM. With the exception of the Up and go test, a higher score indicates a better result. a. The linear mixed model analysis was carried out with the time*treatment interaction. b. The linear mixed model analysis was carried out without the time*treatment interaction. Abbreviations: CPC: citrus and pomegranate complex; SFT: senior fitness test. * p < 0.05.

For the other physical fitness tests, no time*treatment interaction was observed. For the Chair Stand Test, Biceps Curl Test, and Up and Go test, a significant effect of time was observed (Chair Stand Test: Δ = 0.3 repeats, p = 0.034; Biceps Curl Test: Δ = 0.6 repeats, p = 0.009; Up and Go test: Δ = 0.1 s, p = 0.006), with both outcomes improving over time. No significant time or treatment effect were observed for the remaining physical fitness parameters.

### Quality of Life Questionnaire

Results from the WHO-QOL-100 questionnaire were reported in Table 2. For the overall score and the six domain scores, no significant time*treatment effects were observed. However, a trend for a time*treatment interaction on the psychological function domain was found (p = 0.061). To further investigate the origin of this effect, the five facets that make up psychological function were analyzed separately: positive feelings; thinking, learning, memory, and concentration; self-esteem; bodily image and appearance; and negative feelings. Results can be found in Table 3. A significant time*treatment effect (p = 0.042) was observed for thinking, learning, memory, and concentration, showing improved scores after supplementation with CPC, compared to placebo. For the other facets, no time*treatment interaction or main effect of time or treatment was observed.

**Table 2:** Outcomes of the QOL-100 questionnaire after four weeks of CPC or placebo supplementation^1^

**Quality of Life (T-scores)**	**CPC (n=36)**	**Placebo (n=36)**	Time*treatment interaction	**Main effect of time**	**Main effect of treatment**
Overall score			0.409^a^	0.313^b^	0.666^b^
Baseline	81.4 ± 2.6	81.6 ± 2.4			
4 weeks	81.3 ± 2.4	79.9 ± 3.0			
Physical capability			0.588^a^	0.538b	0.447b
Baseline	73.7 ± 2.2	75.1 ± 2.1			
4 weeks	73.7 ± 2.3	74.2 ± 2.3			
Psychological function			0.061^a^	0.589b	0.279b
Baseline	71.5 ± 2.1	72.0 ± 2.1			
4 weeks	73.0 ± 2.2	71.3 ± 2.2			
Level of independence			0.843^a^	0.860^b^	0.098^b^
Baseline	85.0 ± 2.0	83.6 ± 1.9			
4 weeks	84.7 ± 2.0	83.6 ± 2.1			
Social relationships			0.379^a^	0.713^b^	0.140^b^
Baseline	74.7 ± 2.3	76.7 ± 2.2			
4 weeks	75.7 ± 2.3	76.3 ± 2.3			
Environment			0.280^a^	0.588^b^	0.951^b^
Baseline	78.8 ± 1.9	79.4 ± 1.8			
4 weeks	79.1 ± 1.8	78.5 ± 2.0			
Spiritual			0.660^a^	0.589^b^	0.686^b^
Baseline	53.3 ± 2.9	53.3 ± 3.7			
4 weeks	53.5 ± 3.2	55.0 ± 3.1			


1. Data are presented as actual mean ± SEM. a. The linear mixed model analysis was carried out with the time*treatment interaction. b. The linear mixed model analysis was carried out without the time*treatment interaction. Abbreviations: CPC: citrus and pomegranate complex; QOL-100: quality of life questionnaire 100 items.

**Table 3:** Outcomes of the QOL-100 Psychological Function facets after four weeks of CPC or placebo supplementation^1^

**Psychological Function (T-scores)**	**CPC (n=36)**	**Placebo (n=36)**	Time*treatment interaction	**Main effect of time**	**Main effect of treatment**
Positive feelings			0.103^a^	0.745^b^	0.785^b^
Baseline	70.1 ± 2.0	71.5 ± 2.1			
4 weeks	72.2 ± 1.9	70.1 ± 2.0			
Thinking, learning, memory, concentration			0.042^a^*	0.286^a^	0.042^a^*
Baseline	67.9 ± 3.0	67.5 ± 2.9			
4 weeks	71.2 ± 3.2	66.5 ± 3.0			
Self-esteem			0.565^a^	0.728^b^	0.097^b^
Baseline	66.8 ± 2.1	65.6 ± 2.3			
4 weeks	67.5 ± 2.3	65.5 ± 2.6			
Bodily image and appearance			0.152^a^	0.858^b^	0.494^b^
Baseline	77.3 ± 2.8	79.5 ± 2.8			
4 weeks	78.8 ± 2.9	78.3 ± 2.6			
Negative feelings			0.514^a^	0.512^b^	0.385^b^
Baseline	24.5 ± 2.6	24.1 ± 2.4			
4 weeks	26.0 ± 3.2	24.1 ± 2.7			


1. Data are presented as actual mean ± SEM. a. The linear mixed model analysis was carried out with the time*treatment interaction. b. The linear mixed model analysis was carried out without the time*treatment interaction. Abbreviations: CPC: citrus and pomegranate complex; QOL-100: quality of life questionnaire 100 items. * p < 0.05.

### Blood biomarkers

Levels of the blood biomarkers before and after four weeks of supplementation with CPC or placebo were reported in Table 4. The time*treatment interaction for MDA was significant (p = 0.033), showing decreased levels of MDA after CPC supplementation, compared to placebo. For TEAC and NO3, no significant interaction was observed. A significant effect of time was found for TEAC (p = 0.017).

**Table 4:** Levels of blood biomarkers after four weeks of CPC or placebo supplementation^1^

**Blood Biomarkers**	**CPC (n=36)**	**Placebo (n=36)**	Time*treatment interaction	**Main effect of time**	**Main effect of treatment**
TEAC (mmol Trolox equivalent/L)			0.511^a^	0.017^b^*	0.676^b^
Baseline	0.29 ± 0.02	0.28 ± 0.02			
4 weeks	0.27 ± 0.02	0.27 ± 0.02			
MDA (*µ* mol/L)			0.033^a^*	0.660^a^	0.541^a^
Baseline	2.00 ± 0.17	1.69 ± 0.11			
4 weeks	1.72 ± 0.08	1.87 ± 0.11			
NO3 (*µ* mol/L)			0.249^a^	0.981^b^	0.077^b^
Baseline	59.8 ± 5.8	54.7 ± 7.6			
4 weeks	67.3 ± 11.2	47.0 ± 3.9			


1. Data are presented as actual mean ± SEM. a. The linear mixed model analysis was carried out with the time*treatment interaction. b. The linear mixed model analysis was carried out without the time*treatment interaction. Abbreviations: CPC: citrus and pomegranate complex; MDA: malondialdehyde; NO3: nitrate; TEAC: Trolox equivalent antioxidant capacity; * p < 0.05.

## Discussion

In this study, the effects of CPC on physical fitness and mental wellbeing were evaluated in older adults. After four weeks of supplementation with CPC, handgrip strength significantly improved, as compared to placebo, which deteriorated. Moreover, self-evaluated psychological function showed a trend for improvement after CPC supplementation. This was supported by a significantly higher score for the CPC group in the thinking, memory, learning, and concentration facet, as compared to the placebo group. Lastly, a significant decrease in MDA was observed after CPC supplementation. No significant differences for the senior fitness test, the other QOL domains and blood parameters were observed between intervention and placebo.

This study was the first to determine the effects of a combined citrus and pomegranate complex on physical fitness in an older study population. Outcomes related to physical fitness included the handgrip strength test and the senior fitness test battery. Improved handgrip strength has previously been associated with reduced all-cause mortality, as well as with increased cognitive function, mobility, and functional status (30, 31). Moreover, in a cross-sectional study including over 68,000 participants, handgrip strength was positively associated with consumption of various food items in both men and women, and specifically fruit and vegetables in women (32). The values for handgrip strength observed in this study were comparable to normative values of the UK biobank, as well as a cohort from the United States within the same age group (33, 34). Only two previous studies have investigated the effects of a polyphenol-rich intervention on handgrip strength, both of which were performed in male adults (35, 36). Supplementation with grape juice in male volleyball athletes did not result in improved handgrip strength (35), while a significant improvement in handgrip strength 24 hours after intense resistance exercise was observed in male adults (36). Similar to our study, several polyphenol-rich intervention studies using different isometric strength tests have observed improvements in recreationally active males and older subjects (37, 38, 39). Here, we observed a slight increase in the intervention group as well as a decrease in the placebo group. Mitchell et al. (40) have shown that ageing results in muscle strength loss of 2.5–4% per year. However, the decrease in muscle strength observed in the placebo group is higher than expected based on ageing alone. Therefore, other underlying lifestyle factors such as diet and exercise may have been involved in this process. During the study, changes in these factors were not recorded, and participants were asked to refrain from citrus flavonoids. Consequently, the observed deterioration of muscle strength in the control group may have been the result of changes in lifestyle factors, which was attenuated by the CPC intervention.

In contrast, we did not observe significant improvements on the senior fitness test battery in the present study. Several studies have reported significant improvements in the 6MWT and the up and go test in (pre)frail and sarcopenic older adults and patients with peripheral artery disease (38, 41). The difference in health status of the participants (i.e. healthy older adults vs. (pre)frail older adults) could explain the contrasting results. Moreover, in an individualized nutritional intervention in (pre)frail older adults, 10m gait speed was significantly improved after a six month intervention of individualized nutrition, but not after only one month (42). Therefore, the senior fitness test might not be sensitive enough to observe changes in a four week dietary intervention study.

A potential mechanism that might be associated with the improvement in handgrip strength is improved mitochondrial function. With age, the number of mitochondria reduces and functionality decreases, which results in reduced ATP production, mitochondrial biogenesis, imbalanced fusion/fission, impaired mitophagy, and increased ROS production (43). This mitochondrial dysfunction has been shown to be a main contributor to the development of sarcopenia (44, 45). Recently, Migliavacca et al. (47) have provided evidence for a fundamental role of mitochondrial metabolism in skeletal muscle mass and function in elderly. However, based on this mechanism, an effect on walking distance would also be expected. For several dietary polyphenols, a role for mitochondrial metabolism and biogenesis in human health has been established [48]. For hesperetin, the aglycone of hesperidin, increased intracellular ATP production and mitochondrial spare capacity has been observed in vitro (49). Furthermore, neohesperidin, a glycoside of hesperetin, has shown to improve mitochondrial biogenesis in high fat diet fed mice (50). However, to our knowledge, this has not yet been confirmed in humans in vivo.

In this study, facets within the self-evaluated quality of life domain psychological function were improved after supplementation. As quality of life is an important factor of successful ageing, improved psychological functioning is of increasing relevance (51). In line with these findings, in various animal experiments, beneficial effects of hesperidin on depressive disorders, anxiety disorders, and stress were observed (52, 53, 54). Moreover, a review by Hajialyani et al. (55) has reported beneficial effects of hesperidin on neurodegenerative diseases both in animals and human studies. More specifically, a human study evaluating effects of a flavanone-rich orange juice in healthy older adults reported significant effects on global cognitive function (56).

The improvement in hand grip strength and quality of life might be the result of antioxidant properties and improvement of endothelial function. An improvement in antioxidant status during ageing could prevent the negative age-related effects of oxidative stress (57), and a link between polyphenol supplementation, exercise performance, and oxidative stress has already been established (58). Nitrate is considered to be an important signaling molecule involved in endothelial function by causing vasodilation in smooth muscle cells, thereby improving blood flow (59). Consequently, we analyzed TEAC and MDA as measures for oxidative stress, and NO3 as a measure for endothelial function. For TEAC, we did not observe a significant effect of CPC, while MDA decreased after CPC supplementation compared to placebo. In line with this observation, previous studies showed decreased MDA levels after cocoa flavonoid supplementation in older subjects (38) and after hesperidin supplementation in type 2 diabetes patients (60), indicating lowered oxidative stress. Lastly, we observed a slight increase in NO3, even though this did not reach significance. A higher potential to produce nitrite could contribute to enhanced blood flow and improved exercise capacity (61). In vitro studies have shown that exposure to hesperetin increases nitric oxide production in endothelial cells (62), and human in vivo studies have shown that hesperidin and punicalagin supplementation improve endothelial function (63, 64, 65).

A limitation of this study is that no skeletal muscle biopsies were collected. Consequently, effects of CPC supplementation on mitochondrial function could not be investigated. Moreover, the included study population was relatively healthy (BMI 18–28kg/m^2^, normal blood pressure, no cardiovascular disease, non-smokers), which might not be an accurate representation of this age group, as no significant effects on the senior fitness test were observed. For this population, the exercise battery may not have been strenuous enough to sufficiently challenge the participants, in order to detect small dietary intervention-induced improvements in performance capacity. Alternatively, it could be possible that the significant effect on hand grip strength was due to chance, as the other parameters were not significantly different between groups. Lastly, the study was not powered to determine differences between men and women, which could be of interest for future research as gender differences might affect physical activity levels. For a follow up study design, we suggest including physical fitness tests that are considered to be more challenging and collecting muscle biopsies to study potential underlying mechanisms.

In conclusion, this study shows that four weeks of daily CPC supplementation can significantly improve handgrip strength in healthy older adults. Furthermore, self-evaluated measures within the domain of psychological function (QOL) were significantly higher in the CPC group compared to placebo. Finally, blood levels of MDA were significantly decreased after CPC supplementation. Further research should focus on the mechanisms associated with the improvement in physical performance.
